# Determination of the Maximum Velocity of Filaments in the *in vitro* Motility Assay

**DOI:** 10.3389/fphys.2019.00289

**Published:** 2019-03-27

**Authors:** Nasrin Bopp, Lisa-Mareike Scheid, Rainer H. A. Fink, Karl Rohr

**Affiliations:** ^1^Biomedical Computer Vision Group, BioQuant Center and IPMB, University of Heidelberg and DKFZ, Heidelberg, Germany; ^2^Medical Biophysics Unit, Medical Faculty, Institute for Physiology und Pathophysiology, University of Heidelberg, Heidelberg, Germany

**Keywords:** myosin, actin, *in vitro* motility assay, filament, velocity, signal reconstruction, code:R

## Abstract

The *in vitro* motility assay (IVMA) is a powerful tool commonly used in basic muscle research and for drug screenings with the aim to find treatment options for neuromuscular disorders. In brief, the sliding movement of fluorescence-labeled actin filaments on myosin motor proteins is recorded, and the sliding velocity is analyzed via image analysis methods. Due to low signal-to-noise ratios and large variability in the velocity signal, accurate determination of the maximum sliding velocity is challenging. We introduce a new method and software program named Actin Phase Velocity (ActiPHV). The method extracts the maximum velocity from filament tracking data. Based on simulated and real reference data we show that our method yields a higher accuracy compared to previous methods. As a result, our method enables enhancing the sensitivity of the IVMA to better exploit its full potential.

## 1. Introduction

**Figure 1:**
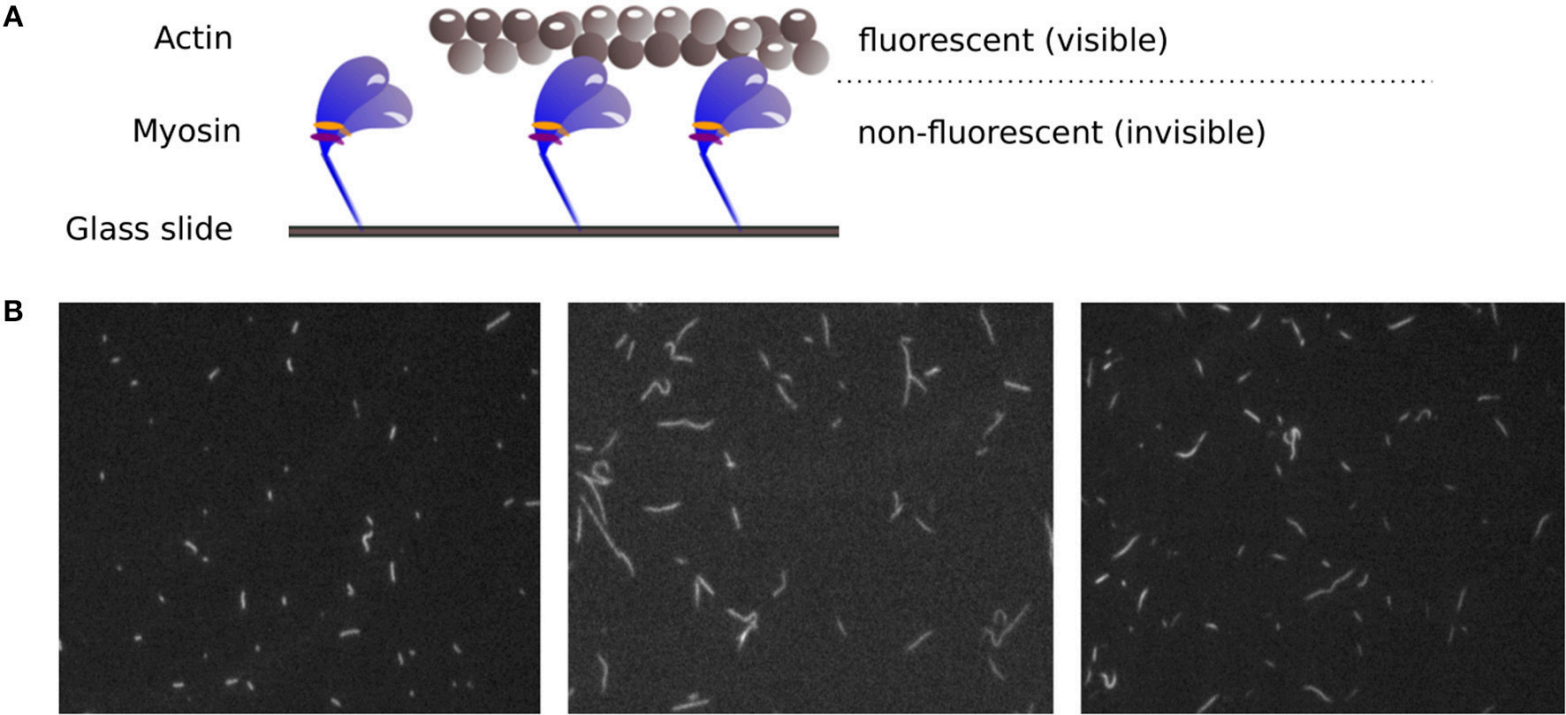
**(A)** Principle of the IVMA. Myosin motor proteins, which are fixed on a glass slide, move fluorescently labeled actin filaments forward. The movement of the actin filaments can be observed, while the myosin proteins remain invisible. **(B)** Example IVMA fluorescence microscopy images.

After years of research on muscle function, there are still muscular disorders that cannot be cured yet. Whereas, the main function of contractile proteins actin and myosin are known, some aspects of how the muscle function is modulated are not completely understood. Mutations of regulatory proteins or myosin light chains are associated with (cardio) myopathies. However, functional outcomes of these mutations need to be characterized. Therefore, it remains an important issue to investigate muscle function on the molecular level. The *in vitro* motility assay (IVMA) (Kron and Spudich, 1986) is a standardized test system used to investigate the interaction between actin and myosin, the two main proteins responsible for muscle contraction. Therein, myosin motor proteins that are fixed on a glass slide transport fluorescently labeled actin filaments, as shown in Figure 1A. Under a fluorescence microscope, the movement of the filaments can be observed. The actin sliding velocity provides indirect information about myosin properties and catalysis rate. The sliding velocity of actin depends particularly on the myosin-ATPase rate, the myosin type, and the myosin density on the slide (see, e.g., Yengo et al. (2012)). It has recently been shown that the IVMA time scale of actin-myosin interaction (with, e.g., myosin II motor protein cycle times in the range of 150 ms) is mainly determined by the myosin functioning as an ensemble (Rastogi et al., 2016). The assay is performed in a large number of variations using proteins from different animals (Höök et al., 1999; Elangovan et al., 2012; Scheid et al., 2017). It can be used to selectively study myosin isoforms and actin mutants, and for drug screenings to find candidates for the treatment of skeletal and cardiac muscle diseases. For example, only recently the role of the essential myosin light chain in heart function was further clarified using IVMAs (Scheid et al., 2016). For all variations of the IVMA, the actin filament sliding speed is the common and most important readout.

While most IVMA analysis algorithms only determine the mean filament velocity in an image sequence, observations on regulated IVMAs can highlight the relevance of analyzing maximal sliding speeds. Gordon and colleagues found faster sliding speeds of reconstituted thin filaments compared to actin filaments in the presence of calcium (Gordon et al., 1997). Thus, analysis of the maximal sliding speed is an important readout for regulated IVMAs and provides biologically relevant information on the underlying actin-myosin interaction.

However, the accurate analysis of filament velocities in the IVMA is complicated by several factors. The acquired spatially and temporally high resolved image sequences of the IVMA, as shown in Figure 1B, have a generally low signal-to-noise ratio. In addition, there is a large variability in image contrast within and between image sequences. The filament movement is unidirectional, but the movement paths are randomly distributed and contain irregularities, causing filament crossings, filament breaks, circular movements, sudden stops, and periods of slower filament movement. For analyzing IVMA images, different methods have been developed. Most of them are based on particle tracking algorithms with preceding segmentation by automatic adaptive thresholding (e.g., “FAST” software) (Aksel et al., 2015) or manual threshold adjustment by the user (e.g., “Diatrack” software) (Vallotton and Olivier, 2013; Vallotton et al., 2017). Due to the highly variable image quality, segmentation without user-intervention often leads to unsatisfactory results. In automatic tracking methods, the filament centroids are usually used to determine the position. Since the velocity is calculated by the filament displacement divided by time, inaccuracies in determining the position contribute to the noise in the velocity signal, which complicates the determination of the maximum velocity. One approach that does not require segmentation is the optical flow-based structure tensor method (Uttenweiler et al., 2000). However, this method only determines the mean velocity, but not the maximum velocity. Aksel et al. (2015) suggested calculating the maximum velocity by using the top 5% fraction of filtered velocities from an image sequence. In our work, we found that this method underestimates the maximum velocity. Therefore, we developed a new method named Actin Phase Velocity (ActiPHV), which yields more accurate results for the maximum filament velocities in the IVMA.

## 2. Methods

Our method ActiPHV (R code available under: https://bitbucket.org/n_Bopp/actiphv) consists of three main steps: First, the filament velocities derived from filament tracking data are preprocessed with a Kalman filter (R-package “FKF,” Kalman,^1960^; Luethi et al.; 2014) for signal reconstruction. Second, different velocity patterns are separated from each other using a split-and-merge “phase identification” method. Third, mean phase velocities are sorted by size and the velocities of the fastest fraction are averaged to determine the maximum velocity.

### 2.1. Velocity Signal Reconstruction

We compared seven different filter methods for the reconstruction of the velocity signal to determine the best method for our application. We used 2D simulated velocity data (330 datasets), which was generated in accordance with real filament velocity data (e.g., constant and periodic velocities with Gaussian and impulsive noise). The real filament velocity data was determined by manual tracking. Compared to real data, simulated data has the advantage that correct ground truth is available. We used the following filter methods: Standard moving average filter, Gaussian filter, median filter, Komogorov-Zurbenko (KZ) filter, Savitzky-Golay (SG) filter (R-package “signal”), Spencer filter (R-package “signal”), and Kalman filter (R-package “FKF”). In brief, the Kalman filter was implemented using the state vector α_*t*_ = (*x*_*t*_, *y*_*t*_, ẋ_*t*_, ẏ_*t*_) representing the 2D position and their derivatives, and the following state transition and measurement equations:

(1) $$\begin{array}{r} \alpha_{t+1}=T_{t}\cdot \alpha_{t}+H_{t}\cdot \eta_{t} \end{array}$$

(2) $$\begin{array}{r} z_{t}=Z_{t}\cdot \alpha_{t}+G_{t}\cdot \epsilon_{t} \end{array}$$

where the state transition matrix *T*_*t*_ propagates the state to the next time step, and *H*_*t*_ · η_*t*_ represents the modeling errors, where η_*t*_ is the Gaussian distributed process noise. The measurement matrix *Z*_*t*_ relates the state to the measurement *z*_*t*_, and *G*_*t*_ · ϵ_*t*_ represents the measurement errors, where ϵ_*t*_ denotes the Gaussian distributed measurement noise. All parameter settings are given in the provided program code.

The performance of the filter methods was determined by

(3) $$\begin{array}{r} R^{2}=1-\frac{SSE_{filter}}{SSE_{total}} \end{array}$$

with the total sum of squared error for the unfiltered (noisy) simulated velocity signal

(4) $$\begin{array}{r} SSE_{total}=\overset{n}{\underset{i=1}{\sum }}{\left(v_{i}-v_{i,true}\right)}^{2}, \end{array}$$

and the sum of squared error for the filtered velocity signal

(5) $$\begin{array}{r} SSE_{filter}=\overset{n}{\underset{i=1}{\sum }}{\left(v_{i,filtered}-v_{i,true}\right)}^{2}. \end{array}$$

*SSE*_*total*_ quantifies the deviation between the unfiltered simulated velocity signal *v* and the known true signal *v*_*true*_. *SSE*_*filter*_ represents the deviation between the filtered velocity signal *v*_*filtered*_ and true signal *v*_*true*_.

### 2.2. Velocity Phase Identification

Due to the random myosin distribution in the IVMA, the actin filament sliding movement is heterogenous, which is reflected by phases of temporarily decreased velocities, phases of zero velocities, and phases with higher velocity variability. To identify velocity phases for determining the maximum velocity, a split-and-merge method was developed. To achieve a reasonable partitioning of the velocity signal, the velocity signal is first preprocessed with a 1D Gaussian filter using a standard deviation of σ = 3 pixels (px) and then split at all local maxima of the filtered signal. After splitting, fractions are merged based on their similarity. Starting from the first fraction, a *t*-test is applied to the signal values in the current and in the subsequent fraction. If the fractions are not significantly different (*p* > 0.05), they are merged and tested against the next fraction. In the case of a significant difference, the fractions stay separated and the algorithm continues by comparing the second fraction with the following fraction until the last fraction is reached. Phases shorter then a minimum length (one second, 10 frames) were excluded from further analysis to remove outliers.

### 2.3. Computation of Maximum Velocity

For the identified phases, the mean velocity is computed. When we apply ActiPHV on filament tracking data of an entire image sequence, we obtain a distribution of phase velocities. This distribution is sorted by decreasing size and the velocities of the fastest phases are summed up until they amount to 10% of the total sum of phase velocities, which is defined as

(6) $$\begin{array}{r} v_{sum}=\overset{N}{\underset{i=1}{\sum }}v_{i} \end{array}$$

Finally, these velocities are averaged to determine the maximum velocity.

### 2.4. Evaluation and Method Comparison

To evaluate the performance of ActiPHV, a 2D simulated image sequence was generated based on measurements in real (experimentally acquired) IVMA images. The image sequence (100 images, 8-bit, 700 × 700 px, background intensity of 80) contains 30 elliptical filaments (image intensity of 130, filament sizes of 17 × 3*px* and 8 × 3*px*) moving with velocities between 2 and 8 px/frame. Each of the filaments changes its velocity twice in the image sequence. Gaussian noise of σ_*n*_ = 15 px was added to the images. The front tips of the filaments in the simulated image sequence were manually tracked using the ImageJ plugin MTrackJ (Meijering et al., 2012) and the tracking data were used as input for ActiPHV.

In addition, the performance was evaluated using 2D real image data acquired with an fluorescence microscope (Olympus IX70, inverted, Xe-light source, 100^*L*^ objective). For this purpose, a reference dataset was generated. In three image sequences (100 images, 100 ms/frame, 16 bit, 640 × 480 px) from one IVMA, the front tips of all visible filaments were tracked manually (MTrackJ, Meijering et al., 2012). Afterwards, ActiPHV was applied to the tracking data. The same image data was also analyzed using the automatic tracking methods Diatrack (Vallotton and Olivier, 2013; Vallotton et al., 2017) and FAST (Aksel et al., 2015). The velocity was computed as the displacement divided by time. As a reference for the maximum velocity, four filaments from the fastest fraction were randomly selected. The filament tip was manually tracked five times and the velocity was derived based on the averaged displacement. Finally, the reference velocity was calculated as the mean velocity of the maximum velocity phases of the four filaments.

## 3. Results

**Figure 2:**
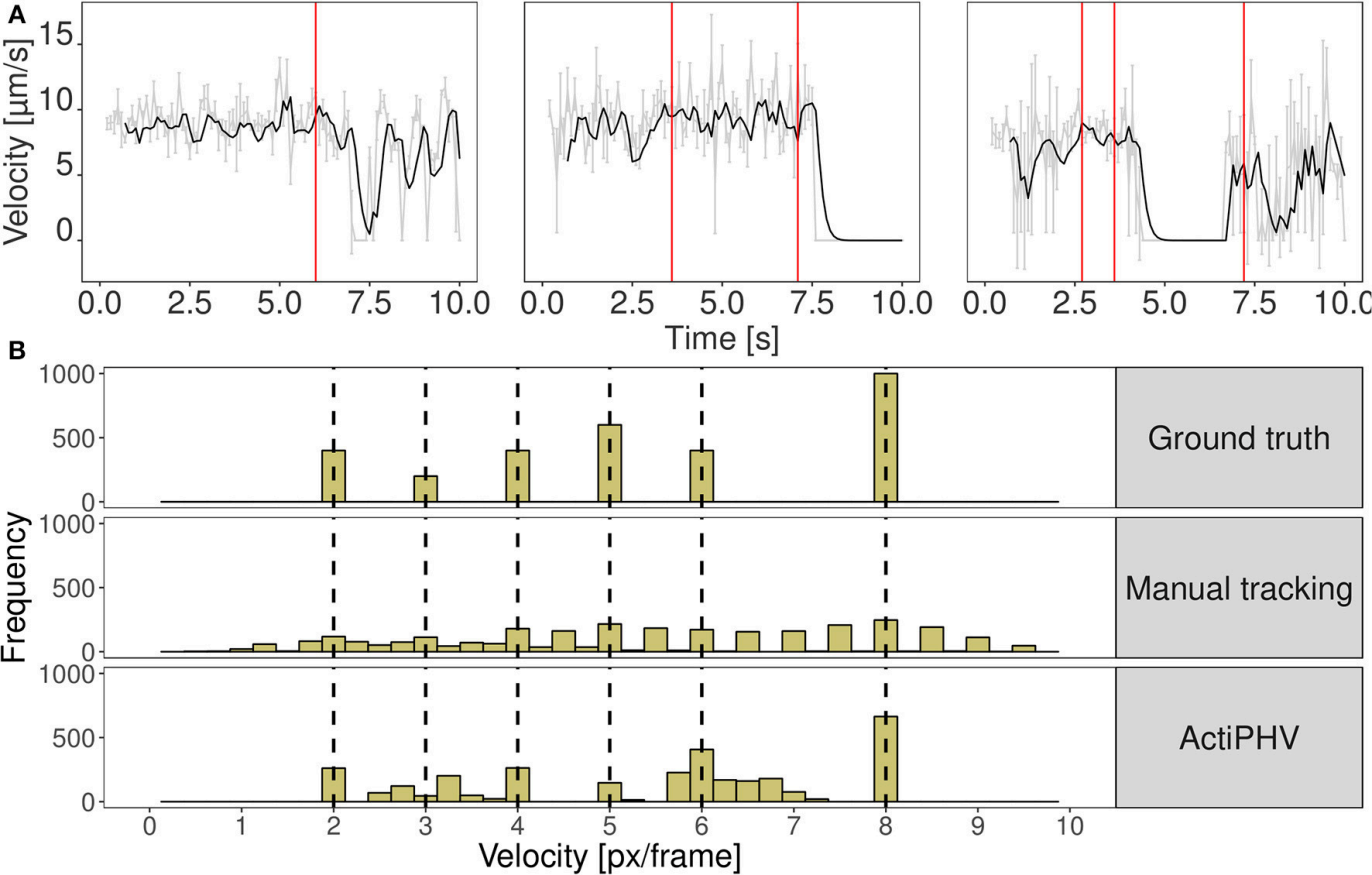
**(A)** Results of ActiPHV for three example filaments that were manually tracked in real image data. The raw signal (gray) is smoothed with a Kalman filter (black) and phases of significantly different velocity patterns are identified (red lines). **(B)** Comparison of velocity distributions for the simulated image sequence. Simulated filaments were tracked manually and ActiPHV was applied to the manual tracking data. Shown are the ground truth velocities (top panel), the velocity distribution after manual tracking (middle panel), and the distribution after application of ActiPHV (bottom panel).

**Figure 3:**
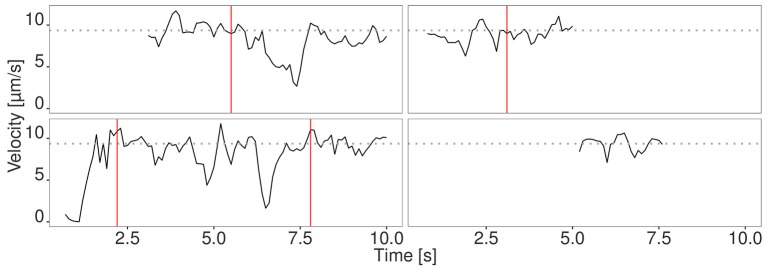
Velocity signal of four randomly selected filaments from the fastest fraction. The filaments move at the maximum velocity (gray dotted line) for a time period longer than 1 s. Different phases are separated by red vertical lines.

We evaluated the seven different methods described in section 2.1 for signal reconstruction to assess their capability to reconstruct the true velocity signal from simulated noisy tracking data. We used 330 simulated tracks and computed as performance measure the mean coefficient of determination $R^{2}=1-\frac{SSE_{filtered}}{SSE_{total}}$, where SSE denotes the sum of squared errors. It turned out that the Kalman filter yielded the best results with *R*^2^ = 0.88±0.05. The other filters yielded the following values: Mean filter: 0.74±0.10, median filter: 0.63±0.07, SG: 0.62±0.13, KZ: 0.80±0.09, Spencer: 0.82±0.10, Gauss: 0.82±0.09. Since the standard deviation for the Kalman filter is low, it can be concluded that signal reconstruction is not only accurate, but also reliable. Therefore, we used Kalman filtering for ActiPHV. After signal reconstruction, phases of significantly different velocity patterns are identified (see Figure 2A). For each phase, the mean velocity, standard deviation, and duration are determined. This results in a distribution of phase velocities when ActiPHV is applied to data of a whole image sequence. Finally, the maximum velocity is computed as the mean velocity of the 10 % fastest fraction of all phase velocities. For performance evaluation, we determined ground truth for the maximum velocity by analyzing the velocity-time courses of randomly selected filaments from the fastest fraction and found a value of $v_{max}=9.7\pm 1.4\frac{\mu m}{s}$ for the reference data (see Figure 3). All these filaments reach the maximum velocity and maintain it over a time period of more than one second (10 image frames).

### 3.1. Evaluation and Method Comparison

In Figure 2B the distribution of ground truth velocities of a 2D simulated image sequence is shown together with the results of manual tracking and ActiPHV. Since manual tracking is error-prone, the velocity distribution is very broad. A better result is obtained using ActiPHV combined with manual tracking. For the real image data, ActiPHV in combination with manual tracking yielded a maximum velocity of $v_{ActiPHV,manual}=9.4\pm 0.2\frac{\mu m}{s}$, which is close to the reference maximum velocity of $v_{max}=9.7\pm 1.4\frac{\mu m}{s}$ (t-test, *p* = 0.09, 95 % confidence level). In comparison, the FAST software (Aksel et al., 2015) yielded a mean velocity of $v_{FAST,mean}=7.7\pm 0.1\frac{\mu m}{s}$ and a maximum velocity of $v_{FAST,max}=8.9\pm 0.2\frac{\mu m}{s}$, which reveals an underestimation. Diatrack (Vallotton and Olivier, 2013; Vallotton et al., 2017) yielded a mean velocity of $v_{Diatrack}=8.9\pm 0.1\frac{\mu m}{s}$, but in combination with ActiPHV we obtain a maximum velocity of $v_{ActiPHV,Diatrack}=9.5\pm 0.4\frac{\mu m}{s}$, which agrees well with the reference maximum velocity. Thus, ActiPHV improves the result compared to existing approaches.

## 4. Discussion

We introduced a new method and software program (ActiPHV) to determine the maximum filament velocity in IVMA microscopy images. From our experiments it turned out that ActiPHV yields a higher accuracy compared to previous methods, which enhances the sensitivity and accuracy of the assay. For simulated data and for real IVMA images, we found that the result of ActiPHV agrees well with the ground truth maximum velocity. We used manual tracking data and automatically determined tracking data. Since manual tracking is very time-consuming and therefore not appropriate for the analysis of IVMA images in daily labwork, accurate automatic tracking methods are essential. Different methods for filament tracking in IVMA images have been introduced. In our work, we used Diatrack (Vallotton and Olivier, 2013; Vallotton et al., 2017) in conjunction with ActiPHV. This tracking method yields good results when detection parameters are adjusted manually for each image sequence. Even though there are partly tracking errors (data not shown), they hardly influence the result of ActiPHV for the maximum velocity, which shows that our method is robust. Thus, our method advances the analysis of IVMA images and enables to better exploit the power of the assay.

## Author Contributions

NB, RF, and KR prepared the concept for this work. NB developed the analysis method ActiPHV. KR supervised the development of the method, while L-MS and RF supported the work from the laboratory point of view by regular review and discussion of intermediate results. In addition, L-MS provided any IVMA images that were required for the development of ActiPHV and the IVMA scheme and images in Figure 1. NB drafted the code article, which was critically revised by all authors.

### Conflict of Interest Statement

The authors declare that the research was conducted in the absence of any commercial or financial relationships that could be construed as a potential conflict of interest.
